# FIB-SEM imaging of carbon nanotubes in mouse lung tissue

**DOI:** 10.1007/s00216-013-7566-x

**Published:** 2014-01-22

**Authors:** Carsten Købler, Anne Thoustrup Saber, Nicklas Raun Jacobsen, Håkan Wallin, Ulla Vogel, Klaus Qvortrup, Kristian Mølhave

**Affiliations:** 1 DTU Nanotech, Technical University of Denmark, Ørsteds Plads 345E, 2800 Kgs. Lyngby, Denmark; 2 DTU CEN, Technical University of Denmark, Fysikvej 307, 2800 Kgs. Lyngby, Denmark; 3 National Research Centre for the Working Environment, Lersø Parkallé 105, 2100 Copenhagen, Denmark; 4 Institute of Public Health, University of Copenhagen, Øster Farimagsgade 5, 1014 Copenhagen, Denmark; 5 Department of Biomedical Sciences, CFIM, University of Copenhagen, Blegdamsvej 3, 2200 Copenhagen, Denmark

**Keywords:** Imaging (NMR microscopy | electron microscopy), Bioanalytical methods, Biological samples, Forensics/toxicology, Nanoparticles/nanotechnology

## Abstract

Ultrastructural characterisation is important for understanding carbon nanotube (CNT) toxicity and how the CNTs interact with cells and tissues. The standard method for this involves using transmission electron microscopy (TEM). However, in particular, the sample preparation, using a microtome to cut thin sample sections for TEM, can be challenging for investigation of regions with agglomerations of large and stiff CNTs because the CNTs cut with difficulty. As a consequence, the sectioning diamond knife may be damaged and the uncut CNTs are left protruding from the embedded block surface excluding them from TEM analysis. To provide an alternative to ultramicrotomy and subsequent TEM imaging, we studied focused ion beam scanning electron microscopy (FIB-SEM) of CNTs in the lungs of mice, and we evaluated the applicability of the method compared to TEM. FIB-SEM can provide serial section volume imaging not easily obtained with TEM, but it is time-consuming to locate CNTs in the tissue. We demonstrate that protruding CNTs after ultramicrotomy can be used to locate the region of interest, and we present FIB-SEM images of CNTs in lung tissue. FIB-SEM imaging was applied to lung tissue from mice which had been intratracheally instilled with two different multiwalled CNTs; one being short and thin, and the other longer and thicker. FIB-SEM was found to be most suitable for detection of the large CNTs (Ø ca. 70 nm), and to be well suited for studying CNT agglomerates in biological samples which is challenging using standard TEM techniques.

**Electronic supplementary material:**

The online version of this article (doi:10.1007/s00216-013-7566-x) contains supplementary material, which is available to authorized users.

**Figure:**
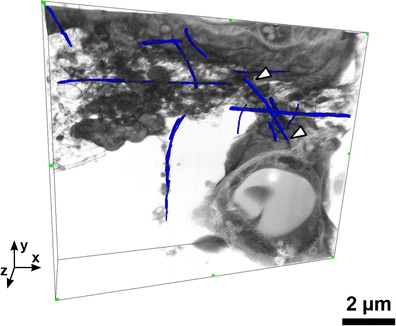
3D FIB-SEM image reconstruction of carbon nanotube (CNT) sample in lung tissue obtained with the double tilted milling method. A few CNTs have manually been traced in the 3D volume, and the white arrowheads point to a single CNT. A – alveole, E – erythrocyte, and P1 – pneumocyte (type 1).

## Introduction

Carbon nanotubes (CNTs) are a very promising nanomaterial in a wide variety of applications due to their excellent mechanical and electrical properties [1, 2]. However, concerns have been raised about safety due to their chemical stability and structural similarity to asbestos fibres. Pulmonary exposure is the exposure route of primary concern both in the working environment and in the general environment. Accordingly, it is important to understand the potential interaction between CNTs and the lung, which is why lung tissue has been chosen for this study. The concerns have been strengthened as pulmonary exposure to CNTs in a number of animal studies has shown a very consistent asbestos-like toxicological response characterised by inflammation, granulomas and fibrosis with low no-effect levels [3–5].

In order to predict the toxicity of CNTs and to make them safe-by-design, it is important to be able to link the toxicity of engineered CNTs to their physical and chemical properties such as length, diameter, coating, charge, and impurities, and to understand how they affect, enter, and eventually locate within the different cell types in the lung.

High-resolution electron microscopy has aided in the understanding of the uptake mechanisms of CNTs [6, 7], which unlike asbestos are able to penetrate and enter cells directly without endocytosis [8]. Additionally, advanced TEM techniques have demonstrated how CNTs can escape endosomal membranes [9] and thereby challenge phagocytic cells in a manner not recognised from asbestos fibre research, as the toxicity of asbestos to a higher degree is caused by “frustrated phagocytosis” [10].

In the bright-field transmission electron microscopy (BF-TEM) imaging mode, agglomerates (non-specifically bound bundles) or even single multiwalled CNTs (MWCNT) have been observed using various in vitro models [9, 11, 12]. Pantarotto et al. studied HeLa cells exposed to CNTs, the TEM images revealed that CNTs were dispersed in the cytosol and appeared absorbed via a non-endocytotic pathway, which was confirmed using endocytosis inhibitors. Both Lee et al. [11] and Al-Jamal et al. [9] studied CNT uptake by macrophages, which mainly revealed CNTs being located in agglomerates within vesicles inside the cells. Interestingly, Al-Jamal et al. noted that 14 days after exposure, TEM images showed that the CNTs were more individually dispersed in the cytoplasm, indicating that the CNTs had escaped the vesicle enclosure [9]. Additionally, Al-Jamal et al. imaged individual CNTs apparently in the process of directly crossing the plasma membrane and showed how the plasma membrane could enwrap single CNTs [9]. TEM imaging has also been performed on in vivo samples [13, 14]. Using light microscopy (LM), Ronzani et al. [13] observed bundles of MWCNTs in alveolar macrophages, which was confirmed with TEM. However, BF-TEM imaging further revealed CNTs in neutrophils and in the mucus layer lining the ciliated epithelial cells, which was not resolved with LM due to the low CNT concentration present in these cell types [13].

BF-TEM has its disadvantages particularly in resolving smaller CNTs, such as single-walled CNTs (SWCNT), from the carbon-rich environment. Especially, Porter and her group have employed materials science TEM techniques such as High Angular Annular dark field TEM (HAADF-TEM), energy filtered TEM (EF-TEM), and TEM tomography, to provide more selective detection of CNTs against a carbon-rich background such as the embedding resin. For example, EF-TEM has been used to create contrast between CNTs and non-stained cells [15]. For a review of these methods, please refer to [7].

A major drawback with TEM when trying to resolve how CNTs enter cells is the loss of the third dimension, as it can be difficult to distinguish between whether CNTs are inserted through the membrane, are membrane bound, or have been dislodged during microtomy and thereby lie on the surface of the TEM section [9]. The issue can in part be circumvented by performing 3D TEM tomography, where a tilt series of TEM images are reconstructed into a 3D representation of the sample [9, 16]. Tomography adds time-consuming complexity to the imaging while still only obtaining data from a very limited volume only about 300 nm thick [16].

Both ordinary TEM and TEM tomography of CNTs in biological samples are prone to preparation artefacts when using ultramicrotomy to cut the ultrathin sections [8, 16]. The hard particles (e.g. CNTs) often cause damage to the diamond knife used in ultramicrotomy as they are not readily cut, instead the particles are often torn from the sections and cause scratches and holes in the section [8, 16, 17].

To circumvent the ultramicrotomy artefacts from TEM preparation, a focused ion beam (FIB) in combination with a scanning electron microscope (FIB-SEM) is an alternative method. The samples for FIB-SEM are processed and stained much in the same manner as embedded TEM samples [18–20], but instead of using a diamond knife an ion beam is used to expose the sample. The ion beam mills through the embedded sample material and uncovers a new surface of the sample which can be imaged with the SEM. FIB-SEM can be operated in an automated mode with sequential milling and image recording. After 3D reconstruction the image stack can provide a larger volume compared to TEM tomography, but with a slightly lower resolution (typically 5–20 nm) compared to TEM (typically 2–5 nm in biological samples) [18, 21]. With FIB-SEM, the orientation of the CNTs in relation to the cell membrane is not critical, as the 3D image data can be reconstructed to a 3D volume with potential isometric resolution. Compared to TEM, the FIB-SEM has the potential to avoid microtomy artefacts and render fast 3D images to uncover the CNT-cell interaction. Even though FIB-SEM has been noted to have great potential for mapping nanoparticles inside tissue and has been used to image MWCNTs in monocyte cells [7], this method has yet to be applied to investigating CNTs in tissue, and tested whether it in fact provides easy and artefact-free volume imaging of CNT exposures.

In this paper, we present TEM images of two types of CNTs in mouse lung tissue and some of the consequences of ultramicrotomy artefacts. In addition, we present SEM images of the microtomed block face, which can be used to localise regions of interest due to protruding uncut CNTs. Subsequently, we study how FIB-SEM can be used to provide images of CNTs in lung tissue, and the artefacts linked to this method. Additionally, we discuss how to limit milling artefacts and introduce a new milling geometry (called double non-tilted milling) to avoid such artefacts. Using two types of CNTs with varying size and stiffness, we illustrate the applicability and critically assess the limitations of this method. We also document how the FIB-SEM can work as a complementary tool to TEM for imaging CNTs in biological samples especially in ‘hot-spot’ regions with high concentration of agglomerated CNTs.

## Materials and methods

### CNTs

**Fig. 1:**
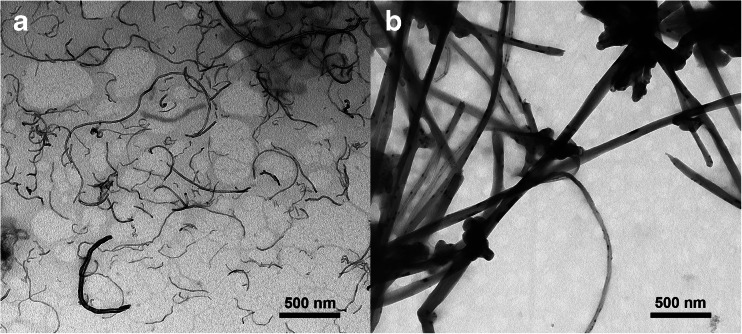
TEM micrographs of the two CNT types used. **a** CNT_Small_. **b** CNT_Large_

A large and a small type of MWCNTs have been used (TEM images in Fig. 1). The small CNT sample was NRCWE-026 (CNT_Small_) from Nanocyl with an average length and width of 850 and 10 nm. The second type (CNT_Large_), was NM-401 which is a test material in the Nanogenotox project [22], and compared to CNT_Small_ these are about five times larger, measuring an average length of 4 μm and having a thickness of about 70 nm. Further information regarding the two types of CNTs is presented in Fig. S1 and Table S1 (Electronic Supplementary Material).

### Mice

Female C57BL/6 mice 5–7 weeks old were obtained from Taconic (Ry, Denmark). The mice were allowed to acclimatise for 2 weeks before the experiment. All mice were fed (Altromin no. 1324, Christian Petersen, Denmark) and allowed water ad libitum during the whole experiment. The mice were group housed in polypropylene cages with sawdust bedding and enrichment at controlled temperature 21 ± 1 °C and humidity 50 ± 10 % with a 12-h light/12-h dark cycle. Female mice were studied at 8 weeks of age. The experiments were approved by the Danish “Animal Experiments Inspectorate” (permit 2010/561-1779) and carried out following their guidelines for ethical conduct and care when using animals in research.

### Preparation of exposure stock and intratracheal instillation of CNTs

CNTs were suspended by sonication in NanoPure water containing 2 % serum collected from C57BL/6 mice. The particle suspensions (3.24 mg/ml) were sonicated using a Branson Sonifier S-450D (Branson Ultrasonics Corp., Danbury, CT, USA) equipped with a disruptor horn (Model number: 101-147-037). Total sonication time was 16 min at 400 W and 10 % amplitude. During the sonication procedure the samples were continuously cooled on ice. The mice were treated with a single intratracheal instillation of 162 μg of CNTs in a 50-μl volume, as previously described [23]. The mice were anesthetised with 4 % isoflurane in the chamber until fully relaxed and 2.5 % during the instillation. Vehicle controls were intratracheally instilled with NanoPure water with 2 % serum sonicated as described for the CNT suspensions. The samples were part of a toxicological study in which three doses were used (18, 54, and 162 μg/mouse). Only the highest dose was chosen for the electron microscopy method development presented in this study. The dose studied (162 μg) corresponds to pulmonary deposition during 32 eight-hour working days at the current Danish occupational exposure level for carbon black (3.5 mg/m^3^) assuming a 10 % deposition rate [4] and a ventilation rate of 1.8 L/h for mice [24]. Clearance of CNTs from lung has a reported half-life of ca. 1 year [5] and therefore we assume that none of the deposited CNTs would be removed within this time frame.

### Lung tissue

Twenty four hours after the intratracheal instillation, mice were anaesthetised by subcutaneous injection of Hypnorm–Dormicum and the mice were bled by cutting the groin. The lungs were fixed in situ, by cannulating the trachea and delivering 2 % glutaraldehyde in 0.05 M cacodylate buffer (pH 7.2) at a constant fluid pressure of 30 cm before the thorax was opened. The fixative was mixed from glutaraldehyde (SPI Supplies #02608) and sodium cacodylate (Sigma-Aldrich #C4945). Thereafter, the lungs were excised and immersed in 2 % glutaraldehyde 0.05 M cacodylate buffer (pH 7.2) and stored refrigerated until further processing.

### Sample treatment

Following fixation, homogeneous looking 1 mm^3^ samples of the alveolar regions of the lung were cut out by a scalpel. The samples were rinsed in 0.15 M phosphate buffer (pH 7.2) and subsequently in 0.15 M sodium cacodylate buffer (pH 7.2) and postfixed in 2 % osmium tetroxide (Polysciences #0972A) and 0.05 M potassium ferricyanide (Sigma-Aldrich #702587) in 0.12 M sodium cacodylate buffer (pH 7.2) for 2 h. Following three rinses with Milli-Q, the samples were en bloc stained with 1 % uranyl acetate (Leica Microsystems, Ultrastain-1) in Milli-Q water overnight at 4 ° C. The samples were dehydrated in ethanol and embedded in Epon according to standard procedures (TAAB Laboratories Equipment, TAAB 812 resin kit) (please refer to the fixation and embedding protocol in Table S2, Electronic Supplementary Material).

To prepare the embedded samples for FIB-SEM, and to allow for a comparison between the information gained from TEM and FIB-SEM, ultrathin sections (80 nm) were cut with an ultramicrotome (Leica Ultracut UCT). The diamond knife angle was 6°, while the cutting speed was set to 1.5 mm/s. The sections for TEM were post-stained with uranyl acetate and lead citrate (Leica Microsystems, Ultrastain-2). Following ultramicrotomy, the exposed surfaces were ready for FIB-SEM imaging. TEM imaging was performed on a CM 100 BioTWIN from Philips operated at 80 kV.

### FIB-SEM

**Fig. 2:**
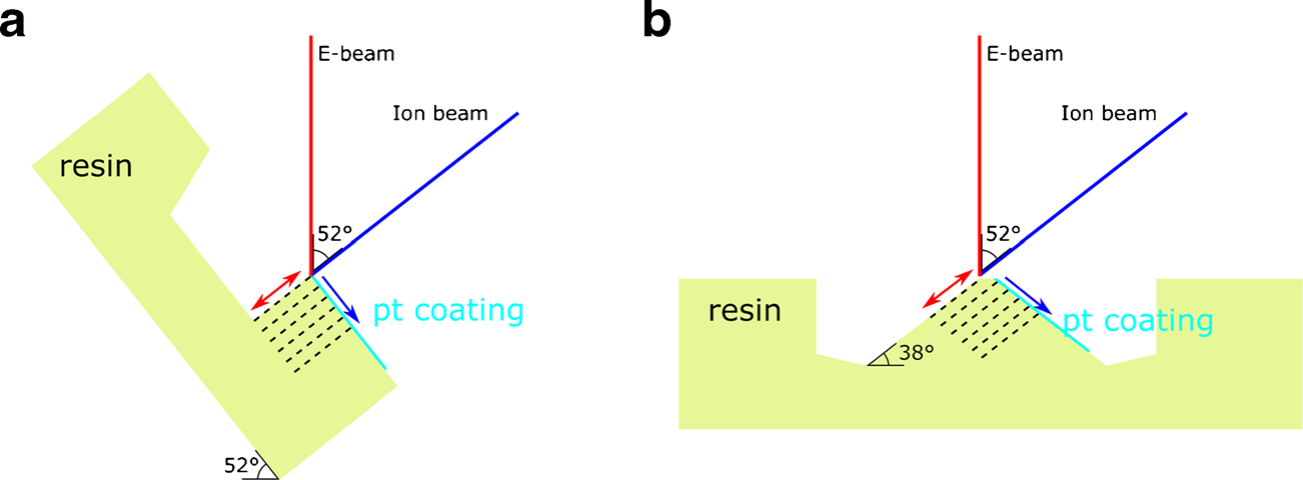
Schematic of the milling geometries used. **a** Standard milling approach where the sample is tilted 52°. **b** The double non-tilted milling method, where the milling is performed without tilting the sample and a wedge is created which has two FIB polished surfaces

Following microtomy, the Epon block samples were mounted with conductive silver paste (EMSdiasum, 12686-15) on SEM stubs and sputter coated with gold. Imaging was performed in high vacuum, and both an in-lens and a designated backscatter detector were used on two different FIB-SEM systems: FEI Helios and FEI Quanta FEG 3D. The Helios in-lens system, with immersion mode operated in either SE or BSE mode, had a higher ultimate resolution than the Quanta FEG 3D FIB-SEM with the designated backscatter detector (vCD—low *v*oltage high *c*ontrast *d*etector). In return, the Quanta FEG 3D detector is more sensitive to backscattered electrons and as the contrast in the sample stems from inelastic scattering on electrons on the heavy metal staining (Z-contrast) [7, 18] the Quanta FEG 3D in our case provided images with better contrast. In SEM, regions of interest with lung tissue and neighbouring protruding CNTs were located with high acceleration voltages (30 kV) for the maximum penetration depth to visualise tissue within the resin block. The angle between the electron- and ion beam was 52° and FIB milling was performed using a 30 kV Ga-ion beam with beam currents ranging from 0.44 to 7 nA during fine- and rough milling, respectively (Fig. 2a). SEM images of the embedded CNTs were obtained using low acceleration voltages (2–5 kV) and low beam currents (1.4–4 nA) to limit beam damage. Image stacks were processed either according to the tilted- or non-tilted milling approach as previously described [20], and subsequently post-processed in Amira.

To limit milling artefacts for obtaining a 3D FIB-SEM image stack, a new milling strategy was introduced, named double non-tilted milling (Fig. 2b). This involves locating an area of interest, where non-tilted milling is performed in front of the protruding CNTs. Next, the sample is rotated 180° around a vertical axis, and again non-tilted milling is performed in front of the CNTs resulting in a blunt wedge (Fig. 2 and Fig. S2, Electronic Supplementary Material). Now the sample is rotated 180° again to clean up the ‘milling surface’ and deposit a 0.5 to 1 μm thick platinum layer on the ‘top surface’, afterwards ordinary slice-and-view imaging performed.

## Results

### TEM of CNTs in lung tissue

TEM analysis verified that the fixation and embedding had maintained adequate preservation of ultrastructure. The control samples displayed well fixed and stained tissue with preservation of organelles such as lamellar bodies and mitochondria (cf. Fig. S3, Electronic Supplementary Material).

**Fig. 3:**
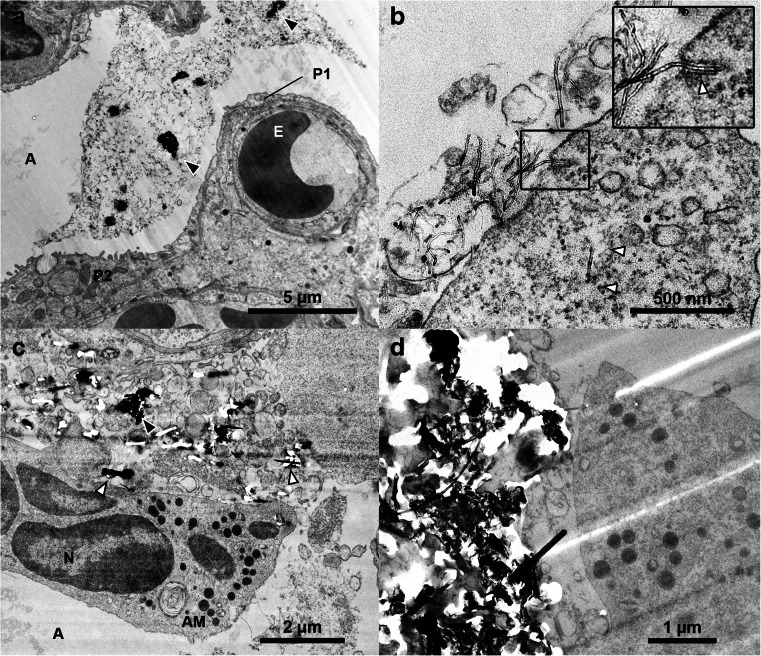
TEM micrographs of lung tissue with CNT_Small_ (**a**–**b**) and CNT_Large_ (**c**–**d**). **a** Overview image of a large agglomeration of CNT_Small_ in the region where black arrowheads highlight very dense CNT agglomerates in the alveolar lumen. The CNTs are causing minor microtomy artefacts (stripes extending from the middle of the image towards the lower right corner). **b** A CNT_Small_ is seen interacting with a cell (*insert*), and a CNT observed freely inside the cytosol (*white arrow*). **c** Overview image showing CNT_Large_ between cells causing major microtomy artefacts. **d** TEM image of a dense agglomeration of CNT_Large_ resulting in holes and stripes in the ultrasection. *A* alveoli, *AM* Alveolar macrophage, *E* erythrocyte, *N* nucleus, *P1* pneumocyte (type 1), and *P2* pneumocyte (type 2). *Black arrowheads* indicate CNT agglomerates, whereas *white arrowheads* indicate single CNTs

As expected, the samples from CNT-treated mice contained more microtomy artefacts than the control sample. Agglomerates of CNT_Small_ tended to give rise to marks (Fig. 3a) caused by the diamond knife failing to cut the CNTs, but the context and ultrastructure of the cells surrounding the agglomerates were still resolvable. Artefacts were especially apparent in the sample with CNT_Large_, see Fig. 3c–d. The CNT_Large_ caused scratches and holes in the sections, in some cases obfuscating the context of the CNTs. TEM images with microtomy artefacts have been observed previously [9, 25–27], generally the artefacts presented in the literature are not as pronounced as in the present study (Fig. 3 and Fig. S3, Electronic Supplementary Material). The reason may be that relatively clear areas are normally chosen for publications and/or that smaller CNTs are studied. However, studying only small CNTs or regions with no sectioning artefacts introduces a bias in the sampling and excludes agglomerates and high concentration ‘hot-spots’ from being studied in the same detail.

Generally, the artefacts comprised of folds, knife marks and holes, but in some instances CNTs were dragged across the sample surface and deposited elsewhere (Fig. S3, Electronic Supplementary Material), as was also documented in [17] for hard particles. In the case of CNT_Large_, artefacts were linked with obvious scratches and drag marks, but besides the marks it was not possible with standard bright-field TEM to document whether the CNT were part of the sample or deposited on top of the section. Clear drag marks were not observed on the CNT_Small_ sample and we found examples of CNTs both inside and outside the cells. In some cases it was clear that the CNT was imaged in place as they would seem to be in the process of penetrating the cell by indentation of the membrane, while others would appear inside a cell without any indication of CNT-cell interaction (Fig. 3b). Accordingly, such images may be interpreted as further proof of CNTs avoiding or escaping the endosomal pathway [9, 12], or alternatively they may represent CNTs which have been dragged and deposited onto the cell leaving an irresolvable dragging path.

### SEM of ultramicrotomed blocks

**Fig. 4:**
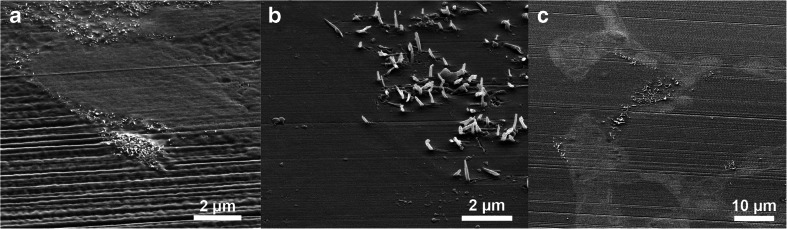
SEM micrographs of the exposed block surface following ultramicrotomy. **a** Image of the CNT_Small_ sample protruding slightly from the microtomed surface. **b** A CNT_Large_ sample after microtomy, where large protruding CNTs are seen which in some cases have left an impression in the Epon block during flattening. **c** Low magnification overview of how lung tissue in close proximity to CNTs can be visualised and later targeted with FIB-SEM

To emphasise the effect that the hard CNTs can have during ultramicrotomy, the blocks were imaged with SEM after ultramicrotomy (Fig. 4). This revealed that both CNT_Small_ and CNT_Large_ remain protruding from the block following sectioning, leaving much of the desired CNTs unsectioned. Similar protrusions have been observed following fracturing CNT composite materials [28, 29]. In the paper by Choi et al., the CNT could in some instances leave indentations of where it had been in the material prior to fracturing. Likewise, we have observed that CNTs can leave an imprint in the Epon layer, which is caused by the diamond knife forcing them to bend and flatten along the surface of the block. Once clear of the diamond knife edge, the stiffness of the CNTs apparently makes them straighten up again.

It should be noted that the microtomy artefacts presented here for the CNT_Large_ samples are quite extreme, but the SEM images can still serve as an example of how much insufficient cutting by the diamond knife can influence what is imaged in the TEM. This could also explain why CNTs are rarely imaged from “atop” having been cut orthogonal to their long axes, as even the small CNTs in this example had protruding CNTs still remaining in the block.

To confirm that the structures protruding from the ultramicrotomed block faces of the embedded lung tissue were indeed CNTs, pure CNTs were embedded and ultramicrotomed (Cf. Fig. S4, Electronic Supplementary Material). Here, the same protruding structures can be seen as in the lung tissue samples. Additionally, we did not observe such structures in the control samples.

### FIB-SEM of CNTs in lung tissue

To avoid the massive ultramicrotomy artefacts especially around ‘hot-spot’ regions which obscure actual cell-CNT interactions, the ion beam of the FIB-SEM can be used to mill through the CNT rich sample.

**Fig. 5:**
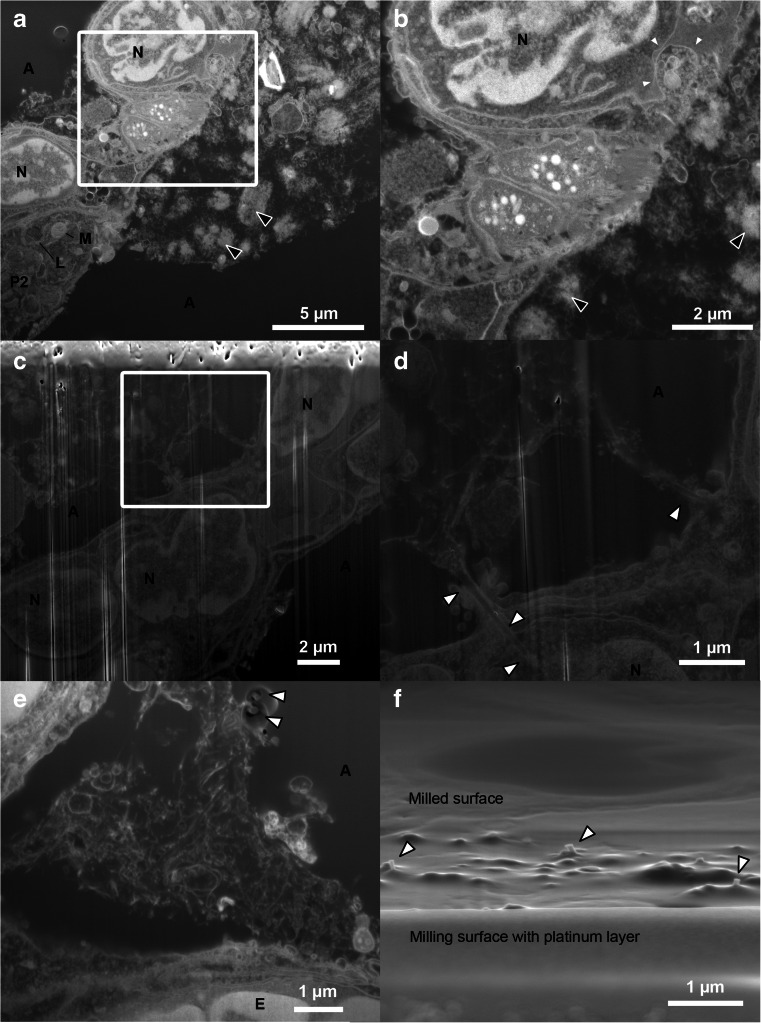
FIB-SEM micrographs of both types of CNTs in the lung samples. **a**–**b** The CNT_Small_ sample imaged with standard milling including a platinum layer, where it can be difficult to discern CNTs from cellular material. *Black arrowheads* mark the likely agglomerations of CNTs not observed in control samples and correlated with CNTs protruding from the surface of the Epon block. One cell appears to have a large invagination, possibly containing CNTs (*small white arrowheads*). **c**–**d** CNT_Large_ samples obtained via standard milling, but without protective platinum layer. Here, the milling artefacts (vertical white lines) caused by surface roughness is clearly seen (especially in **c**). However, the cells and CNTs are still visible, and single CNTs can be found to interact closely with the tissue, but are only in very few cases observed to appear entering the alveolar wall (*white arrowheads* in **d**). **e**–**f** FIB-SEM of CNT_Large_ using the double non-tilted milling approach limiting surface roughness caused artefacts, with *arrowheads* highlighting the protruding CNTs caused by differing milling yields. **f** SEM image obtained from the viewpoint of the ion beam, showcasing that CNTs protrude from the milled surface. *A* alveoli, *E* erythrocyte, *L* lamella body, *M* mitochondrion, *N* nucleus, and *P2* pneumocyte (type 2)

Figure 5 shows recorded FIB-SEM images of CNTs in a toxicologically relevant tissue sample. Previously FIB-SEM images of CNTs in cultured cells have been presented [7]. FIB-SEM images of unexposed mouse lung (control) can be found in Fig. S5, Electronic Supplementary Material. The images clearly show the outline of cells, their nuclei and distinctive organelles. The CNT_Small_ sample (Fig. 5a–b) showcases areas with material outside the cell which looks similar to the agglomerates of CNTs observed in Fig. 3a, but unlike the TEM, SEM does not have the required resolution to distinguish CNTs from other cellular material (also refer to the discussion below and Fig. 7). Due to the resolution of the SEM, it can therefore be difficult to determine whether and how the CNTs and cells interact, but some of the agglomerates appear to be in contact with the cell membrane. The limitations of the FIB-SEM are clearly seen in the highlighted invagination, which could be CNTs in the process of being taken up by the cell (Fig. 5b), but unfortunately the FIB-SEM cannot distinguish these structures from cellular material making confirmation of the observation challenging.

The CNT_Large_ sample (Fig. 5c–f) contains larger structures making them easier to distinguish from the cellular material. CNTs were mostly observed in the intercellular space, an observation confirmed by TEM imaging. However, the FIB-SEM demonstrates that it can produce images of agglomerates of CNT_Large_ with no sectioning artefacts, compared to the shredded ultrasection shown in Fig. 3c–d, which makes it possible to image CNTs apparently penetrating the cell membrane (Fig. 5c–d). The images are not completely artefact-free (Fig. 5c–e), as is evident from vertical white lines (curtaining) and protruding CNTs from the milled surface (Fig. 5f).

Milling artefacts such as curtaining were most pronounced on CNT_Large_ samples and were a result of either the rough milling surface with the protruding CNTs, or the difference in milling yields between the Epon and the CNTs. In areas with extensive protruding CNTs, a smooth milling surface was sought obtained by slow deposition of a thick platinum layer (about 1.5 μm) with the gaseous injection system and the ion beam. The platinum limited the artefacts, but the CNTs underneath created small irregular pockets without platinum thus giving rise to milling artefacts. To provide a smooth milling surface, we introduced a non-tilted milling strategy where the ion beam was used to polish the back and front side of a wedge by rotating the stage (Fig. 2 and Fig. S2, Electronic Supplementary Material). This resulted in an excellent milling surface, albeit it increased the initial milling time significantly.

Both the thick platinum layer and the alternate milling strategy where the milling surface could be polished prior to slice-and-view imaging decreased the milling artefacts, but artefacts originating from the block caused by differing milling yields remained (highlighted by arrowheads in Fig. 5e and Fig. S6, Electronic Supplementary Material). The SEM images of the artefacts caused by insufficient ion milling of the CNTs looks similar to the SEM images by Ke et al. showing CNTs protruding from a surface [30]. To investigate whether it was in fact protruding CNTs, the sample was rotated to image the milled surface (almost) from the point of view of the ion beam (Fig. 5f). This revealed that the newly ion milled surface had small bumps and CNTs protruding from it.

**Fig. 6:**
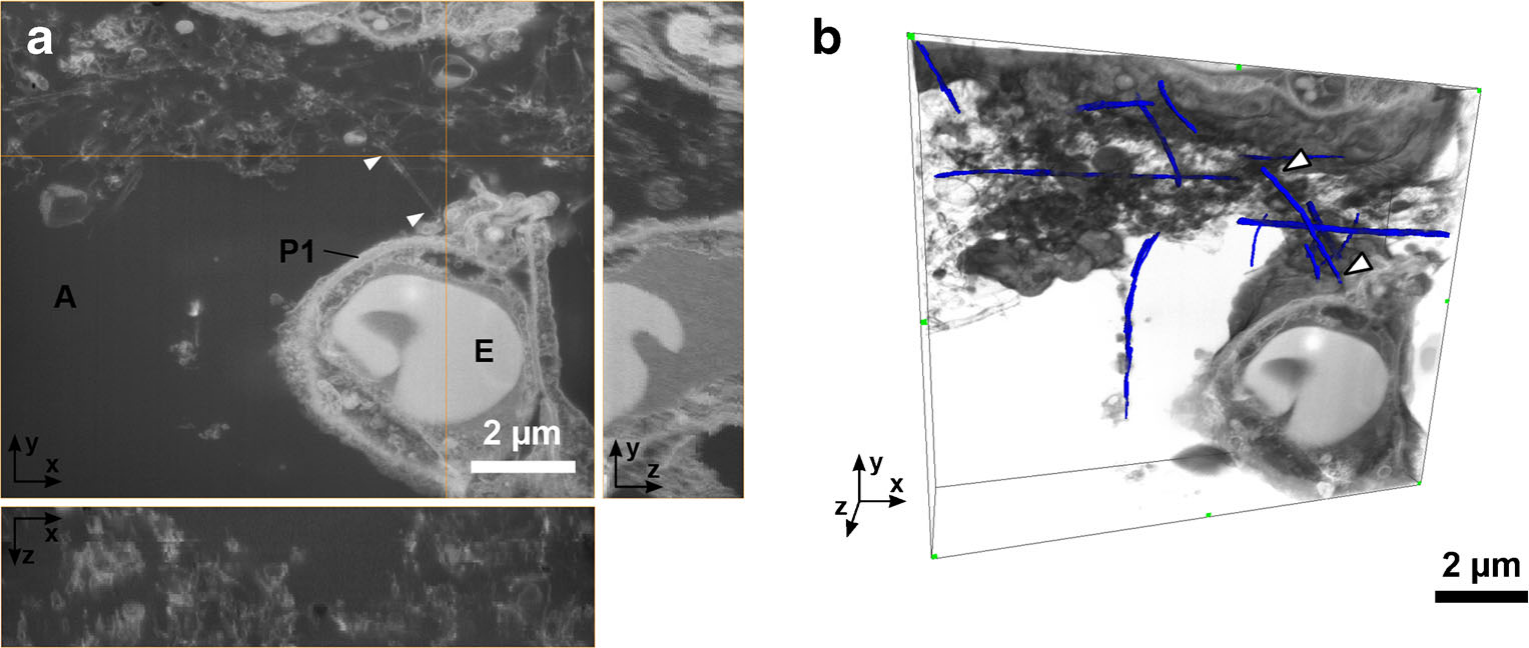
3D FIB-SEM image reconstruction of CNT_Large_ sample obtained with the double non-tilted milling method. **a** Orthogonal *xy*, *xz* and *yz*-views of the stack. **b** 3D view with semi-transparent rendering of the stack. To illustrate the possibility of manually tracing CNTs in 3D a few of the CNTs have been manually coloured blue in Amira. The *white arrows* point to the same CNT in both views. *A* alveoli, *E* erythrocyte, and *P1* pneumocyte (type 1)

FIB-SEM allows volume imaging as illustrated in Fig. 6 and the movie found in the Electronic Supplementary Material (Mov. S1). The 3D stack has been obtained using the double non-tilted milling method (cf. Fig. 2b). The stack of images is aligned and reconstructed as described in [20]. The stack consists of 55 slices which were each 50 nm thick, while the *x*-*y* pixel size was 8.3 nm. This image stack demonstrates one of the strengths of the FIB-SEM, as the 3D information is gathered relatively fast (here in 1 h) and the volume is 2.5 μm thick instead of the 100–300 nm typical for single slice TEM tomography [7]. The TEM slices thereby often only show fragments of the 4-μm-long tube as the section is too thin to contain a long CNT in its entirety. In contrast, we have traced a few of the visible CNTs in the volume to illustrate the capabilities of the FIB-SEM to follow CNTs in 3D (Fig. 6). CNTs were discerned from other tubes and cellular material by following the distinctive parallel lines through the volume (refer to Fig. S7, Electronic Supplementary Material). Single pieces of the same CNTs can thereby be traced across multiple slices and thus follow the entire length of the CNTs (CNT_Large_ mean length 4 μm), instead of only being able to image CNT fragments. Data were obtained from CNT_Large_ samples in areas with a high concentration of agglomerated CNTs, which would have been difficult using standard TEM methods due to microtoming artefacts.

## Discussion

CNTs in tissue can lead to several artefacts when investigated with standard TEM techniques as demonstrated (Figs. 4 and 5). To limit the artefacts, one could seek to optimise the hardness of the embedding medium and the cutting parameters, or even experiment with using a vibrating knife or cryo-ultramicrotome to improve the section quality. Alternatively, some choose to use thicker sections (e.g. 500 nm) at the expense of lower resolution in order to leave a larger volume in which the CNT can remain undisturbed [8]. However, microtomy artefacts are a general issue with particularly large CNTs and many articles contain TEM images of CNTs in cells/tissue with varying degrees of microtomy artefacts which is likely caused by the stiff CNTs [8, 9, 11, 12, 25–27, 31]. Although none of the observed artefacts appear as extreme as we have shown, it is desirable to circumvent these artefacts and also to avoid any sampling bias if we are only able to prepare reasonable images from regions with sparse CNTs for TEM.

We have shown that FIB-SEM can be used to study relatively large CNTs, without physically displacing the CNTs, damaging a diamond knife or causing severe microtomy artefacts. Naturally, FIB-SEM is not artefact-free which can be seen in some of the presented images (e.g. Fig. 5c–f), where curtaining and protruding CNTs can be observed on the milled surface. Ion beam milling artefacts were caused by either surface roughness caused by the protruding CNTs after ultramicrotomy, or by the difference in milling yields between the Epon and the CNTs. To limit the effects of differing milling yields, one could explore the possibility of milling at lower temperatures, adding gases (etchants/water) or changing milling parameters (dwell times, approach and geometry).

**Fig. 7:**
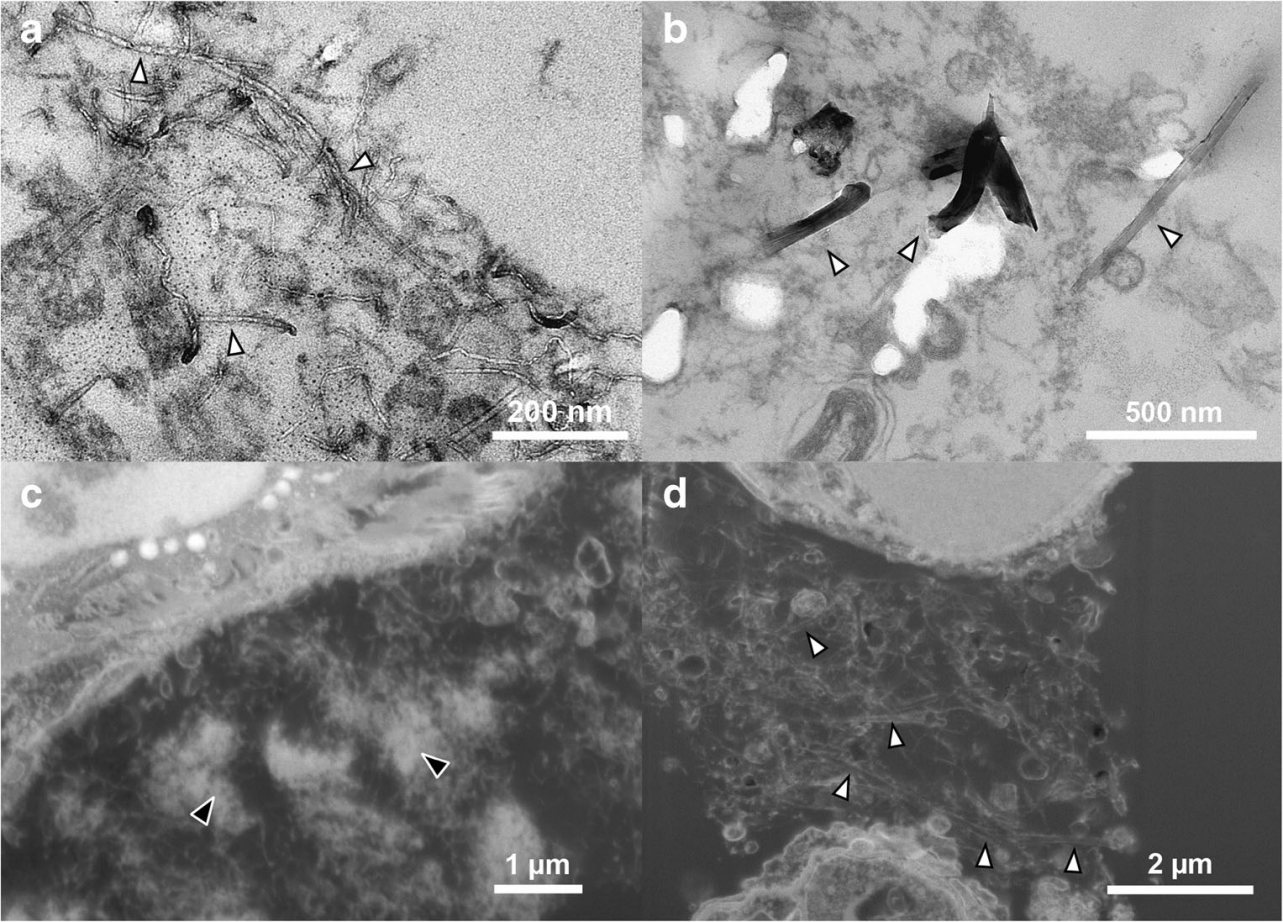
Comparison of the resolution obtainable with TEM and FIB-SEM images of CNTs in lung tissue. **a**–**b** TEM micrographs of the CNT_Small_ and CNT_Large_ sample, respectively. The CNTs can be distinguished from cellular material. **c**–**d** FIB-SEM equivalents of the CNT_Small_ and CNT_Large_ sample, respectively. The micrographs were obtained with the standard milling method including the platinum layer. CNT_Large_ can be visualised as two parallel lines which depending on the imaging method have a weak signal from the centre. But as we approach the resolution limitations of the FIB-SEM the small CNTs are simple lines undistinguishable from cellular material. *White arrowheads* denote single CNTs, and *black arrowheads* agglomerates of CNTs

The images obtained with the FIB-SEM are quite similar to TEM imaging (with inverse contrast), but the FIB-SEM has lower contrast and resolution. This means that in the FIB-SEM images of CNTs in lung tissue, it can be difficult to discern the CNTs from biological material such as stained extracellular matrix proteins, lipid layers, mucus, etc. (cf. Figs. 5 and 7). To illustrate the difference between TEM and FIB-SEM, Fig. 7 shows images of both CNT_Large_ and CNT_Small_ with both methods. In TEM, both types of CNTs can be distinguished from what is considered to be cellular material which has been either excreted from the cells (e.g. from lamella bodies), or is caused by fixation artefacts as described in [17]. The resolution of the TEM even allows for visualisation of CNT_Small_, which is wider and have a different structure than the cellular material (Cf. Fig. 7a), this is not possible in the corresponding FIB-SEM images (Fig. 7c). Using the lamella cut-out method, as demonstrated by Heymann et al. [32], it would be possible to combine the FIB-SEM and TEM, to obtain high-resolution images without the use of a microtome. However, the lamella cut-out method is rather time-consuming and hinders 3D volume imaging.

A drawback of the FIB-SEM is that it is an abrasive method, so there is no way of retrieving an interesting field to obtain a higher resolution image. Likewise, there is little opportunity to characterise interesting sites using methods such as electron dispersive X-ray spectroscopy (EDS), although some groups have managed to perform FIB-SEM and EDS simultaneously [33]. Furthermore, even though large areas can be milled automatically [18] it is very time-consuming and will generally result in an available field of view some 10–100 times smaller than TEM sections. In this paper, the largest milling surface was 30 μm wide whereas the ultrathin sections were approximately 1 mm wide.

In the current study, the interactions are few in number so even though we can find relevant sites, a significant amount of milling time still has to be invested to catch CNTs in the action. For the CNT_Large_ sample alone, ten different sites were investigated and only in two cases, evidence of direct cell-CNT interaction was found and this was for a total slice-and-view time of 20 h (excluding the preparatory work). However, the method itself of locating regions of interest with tissue in close relation to CNTs proved to have an 85 % hit rate when aimed at the regions with protruding CNTs from the sample surface. A higher number of CNT-cell interactions might be observable for longer CNT exposures than those studied in the present study with only 1 day incubation after exposure, as the immune system then would have more time to react. This paper illustrates that FIB-SEM can also be used to image ‘hot-spot’ CNT agglomerates where interaction also occurs.

In order to understand the toxicity of CNTs more information is required on the underlying mechanism by which CNTs cross cellular membranes, but this can be difficult with the limited sample volume of TEM imaging. Thicker sections coupled with 3D tomography can aid in the visualisation [9, 16, 34]. But it has its drawbacks such as a non-isotropic resolution, electron beam caused section shrinkage, and it would still be cumbersome to get nice artefact-free sections containing larger CNTs. To determine whether large CNTs cross cellular membranes or study larger agglomerations of particles, the FIB-SEM has potential because it can be operated in a fairly automated manner. Naturally, the technique is not limited to investigate the toxicity of CNTs only, but could also be applied to many other relevant hard structures that are challenging to microtome [19, 20].

## Conclusion

The aim of this study was to provide an alternative to ultramicrotomy and TEM imaging of CNTs in biological samples. Samples with CNT_Large_ (70 nm wide) caused significant artefacts, especially in ‘hot-spots’ with high CNT concentration, thus potentially leading to sampling bias. Consequently, the feasibility of using FIB-SEM to study CNTs in lung tissue was investigated, and the results compared to standard BF-TEM imaging, as summarised in Table 1.

**Table 1:** Overview of the results from the different methods on the different lung tissue samples from mice exposed to CNT_Small_, CNT_Large_ and control mice instilled with vehicle

	CNT_Small_	CNT_Large_	Control
**TEM of microtomed** **sections**	• Minor ultramicrotomy artefacts • Agglomerates of CNTs resolvable • CNT-cell interaction visible	• Major ultramicrotomy artefacts • Agglomerates of CNTs lost • Can obscure CNT-cell interaction	• Few microtomy artefacts • Excellent ultrastructure • No CNT structures observed
**SEM of microtomed block**	• Small protruding CNTs • 85 % hit rate for regions with CNTs close to tissue	• Large protruding CNTs • 85 % hit rate for regions with CNTs close to tissue	• No CNT protrusions
**FIB-SEM**	• Agglomerates of CNTs visible • CNT-cell interaction not resolvable • Possibility of 3D slice and view	• Agglomerates of CNTs visible • Close CNT-cell interaction observable • Possibility of 3D slice and view	• Acceptable ultrastructure • No CNT agglomerates • Possibility of 3D slice and view
**Conclusion**	• The available FIB-SEM equipment is not suitable for small CNTs • Currently, TEM is the best option.	• FIB-SEM suitable for investigating cellular fate in CNT agglomerates. • Suitable for 3D imaging	• TEM gives the ultimate resolution • FIB-SEM can provide faster 3D imaging

Using FIB-SEM it is very important to be able to localise regions of interest. Following initial sectioning protruding CNTs from the block surface were used to locate regions with CNTs in close proximity to the tissue. CNT_Large_ (70 nm wide) were visualised and could be distinguished from the otherwise carbon-rich environment (embedding material). The FIB-SEMs limited imaging resolution and contrast meant that samples with CNT_Small_ (10 nm wide) could not be distinguished from cellular material.

A 3D FIB-SEM image stack was obtained of the CNT_Large_ sample, by first minimising surface roughness from protruding CNTs. The slice-and-view stack managed to give a 3D image of an agglomerate of CNT_Large_ in the intercellular space with CNTs in some cases slightly touching the cells, which would be troublesome using standard TEM protocols.

In conclusion, we have shown that FIB-SEM can serve as a complementary tool to TEM. It is currently limited to larger CNTs (70 nm wide in our case), but it offers the opportunity to obtain 3D images, without the risk of physically moving CNTs during the sample preparation, and allows the visualisation of the entire CNTs instead of just the fraction present in a 300-nm-thick section.

## Electronic supplementary material

Below is the link to the electronic supplementary material.

ESM 1 (PDF 1.41 MB)

ESM 2 (MPG 6174 kb)
