# Supplementary material for: FIB-SEM imaging of carbon nanotubes in mouse lung tissue
Source: Anal Bioanal Chem. 2014 Jan 22;406(16):3863–73. doi: 10.1007/s00216-013-7566-x (PMC4039996; doi:10.1007/s00216-013-7566-x)
Supplement: Supplementary file 1 — (PDF 1.41 MB) [file 216_2013_7566_MOESM1_ESM.pdf]

Analytical and Bioanalytical Chemistry

Electronic Supplementary Material

# FIB-SEM imaging of carbon nanotubes in mouse lung tissue

Carsten Købler, Anne Thoustrup Saber, Nicklas Raun Jacobsen, Håkan Wallin, Ulla Vogel, Klaus Qvortrup and Kristian Mølhave

**Fig. S1.** TEM images of pure CNTs
**a** and **c** Are TEM images of $CNT_{Small}$, while **b** and **d** are images of $CNT_{Large}$

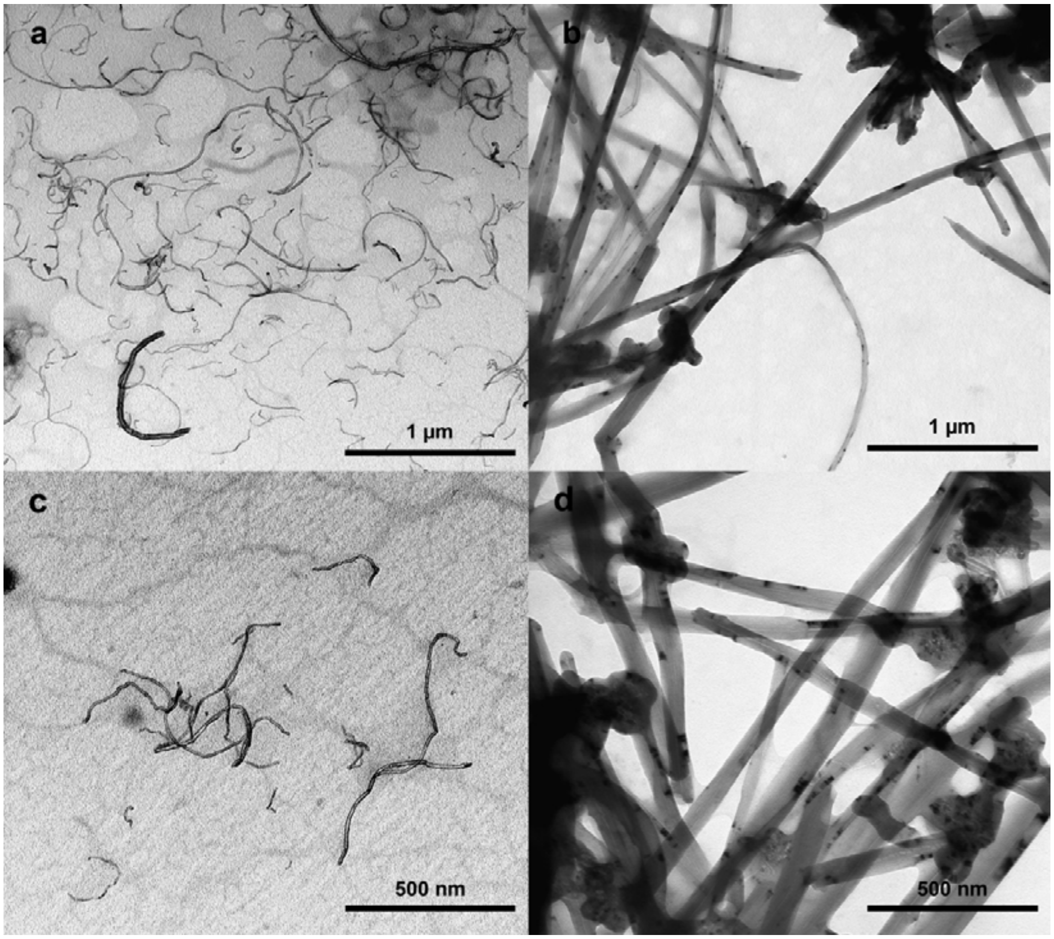

**Table S1.** The dimensions (mean ± SEM) of the two types of CNT as measured in three different manners

'Dried' denotes suspended CNTs which have been allowed to dry out on a grid. 'Lung' refers to TEM measurements of the CNTs in the embedded lung tissue. The final row is the manufacturer's numbers. The results indicate that the widths of the CNTs are represented well in the lung tissue despite the relatively high standard deviation observed for $CNT_{Large}$.

| | **$CNT_{Small}$** (NRCWE-026) | | | **$CNT_{Large}$** (NM-401X) | | |
|---|---|---|---|---|---|---|
| State | Dried | Lung | Manufactor | Dried | Lung | Manufactor |
| Length [nm] | - | - | 850±100 | - | - | 4050±370 |
| Width [nm] | 11±3 | 9±2 | 11±1 | 69±31 | 61±35 | 67±4 |
| N | 30 | 8 | 20 | 30 | 12 | 43 |

**Table S2.** Fixation and embedding protocol

| | | |
|---|---|---|
| Fixation | 2% GA (0.2 M) in 0.05 M cacodylate buffer, pH 7.2 (total 300 mOsm) | Min. 1 day |
| Rinse | 0.15 M phosphate buffer, pH 7.2 | 2 x 30 min |
| Rinse | 0.15 M phosphate buffer, pH 7.2 | 1 x 30 min |
| Post-fix/stain | 2 % $OsO_4$ and 0.05 M $K_3FeCN_6$ in water | 1 hour |
| Rinse | Water | 3 x 15 min |
| En bloc stain | 1%wt Uranyl acetate in water | Overnight |
| Rinse | Water | 3 x 15 min |
| Dehydration | 70% ethanol | 3 x 15 min |
| Dehydration | 96% ethanol | 3 x 15 min |
| Dehydration | 100% ethanol | 3 x 15 min |
| Dehydration | Propyleneoxide | 2 x 15 min |
| Embedding | 1:3 Epon / Propyleneoxide | 45 min |
| Embedding | 1:1 Epon / Propyleneoxide | Overnight |
| Embedding | 3:1 Epon / Propylene oxide | 1 hour |
| Embedding | 100% Epon | Overnight |
| Polymerization | At 60 °C | 48 hours |

Suppliers:

- Glutaraldehyde (CAS# 111-30-8), SPI Supplies (02608-BA)
- Sodium cacodylatetrihydrate (CAS# 6131-99-3), Sigma-Aldrich (C4945)
- Osmium tetroxide (CAS#20816-12-0), Polysciences, inc (0972A)
- Potassium ferricyanide(III) (CAS# 13746-66-2), Sigma-Aldrich (702587)
- Uranyl acetate (CAS# 6159-44-0), Leica microsystems (Ultrastain-1)
- Lead citrate (CAS# 512-26-5), Leica microsystems (Ultrastain-2)
- Propyleneoxide (CAS# 75-56-9), Sigma-Aldrich (110205)
- Epon (TAAB 812 Resin kit), TAAB Laboratories Equipment (T024) via VWR (Bie&Bernsten)

Example image of a CNT filled lung. The lung volume is contained by tying a thread around the trachea:

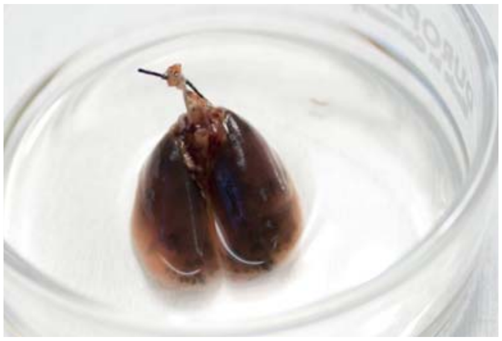

**Fig. S2.** Illustration of the non-tilted milling geometry used to limit milling artefacts

**a** SEM image of the non-tilted milling geometry, where the 'top' surface has been smoothened by the ion beam and a platinum layer has been deposited. **b** schematic of the double non-tilted milling geometry where the 'top' surface has been polished by the ion beam. **c** Schematic of a normal tilted milling geometry.

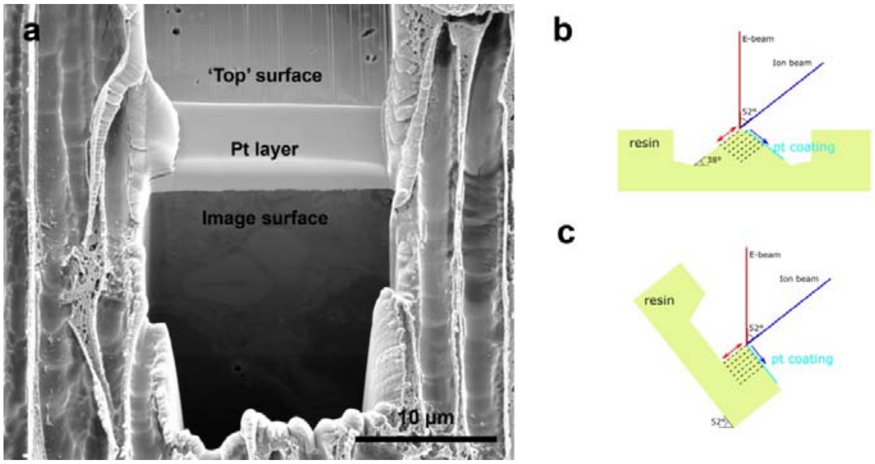


*Ion beam polishing (double non-tilted milling)*

An alternative approach to obtaining a smooth milling surface was to use the ion beam to polish the surface. This involves locating an area of interest, where non-tilted milling is performed in front of the protruding CNTs. Next, the sample is rotated 180 degrees around a vertical axis, and again non-tilted milling is performed in front of the CNTs resulting in a blunt wedge (Fig. 2 and **Error! Reference source not found.**). Now the sample is rotated 180 degrees again to clean up the 'milling surface' and deposit a 0.5 µm to 1 µm thick platinum layer on the 'top surface', afterwards ordinary slice and view imaging performed.

This non-tilted milling approach reduces the intensity gradient effects on the image [1], however, the method does require a two fold increase in rough milling time, but when the preparations have been made the 'slice and view' milling time is similar to the standard method.

[1] Winter DAMD, Schneijdenberg CTWM, Lebbink MN, Lich B, Verkleij AJ, et al. (2009) Tomography of insulating biological and geological materials using focused ion beam (FIB) sectioning and low-kV BSE imaging. Journal of Microscopy 233: 372–383. doi:10.1111/j.1365-2818.2009.03139.x.

**Fig. S3.** TEM images of control- (**a-b**), the $CNT_{Small}$- (**c-d**), and the $CNT_{Large}$ sample (**e-f**)

**a** The control sample show good ultrastructure with visible mitochondria and clearly defined nuclei and cellular walls. **b** A close view of a type II pneumocyte in the alveolar lining containing mitochondria and characteristic lamellar bodies. **c** Close view of a cluster of CNTs in the $CNT_{Small}$ sample showing minor striping artefacts. **d** TEM image of a cluster of small CNTs, again showing clear striping artefacts, but also a hole in the film caused by the very dense region of CNTs. **e** A deployed alveolar macrophage interacting with the CNTs, but the large CNTs cause massive microtomy artefacts with heavy striping and holes. **f** image showing clear drag marks after displacement of the CNT. A – alveole, L – lamella body, M – mitochondrion, N – nucleus, and P2 – pneumocyte (type 2).

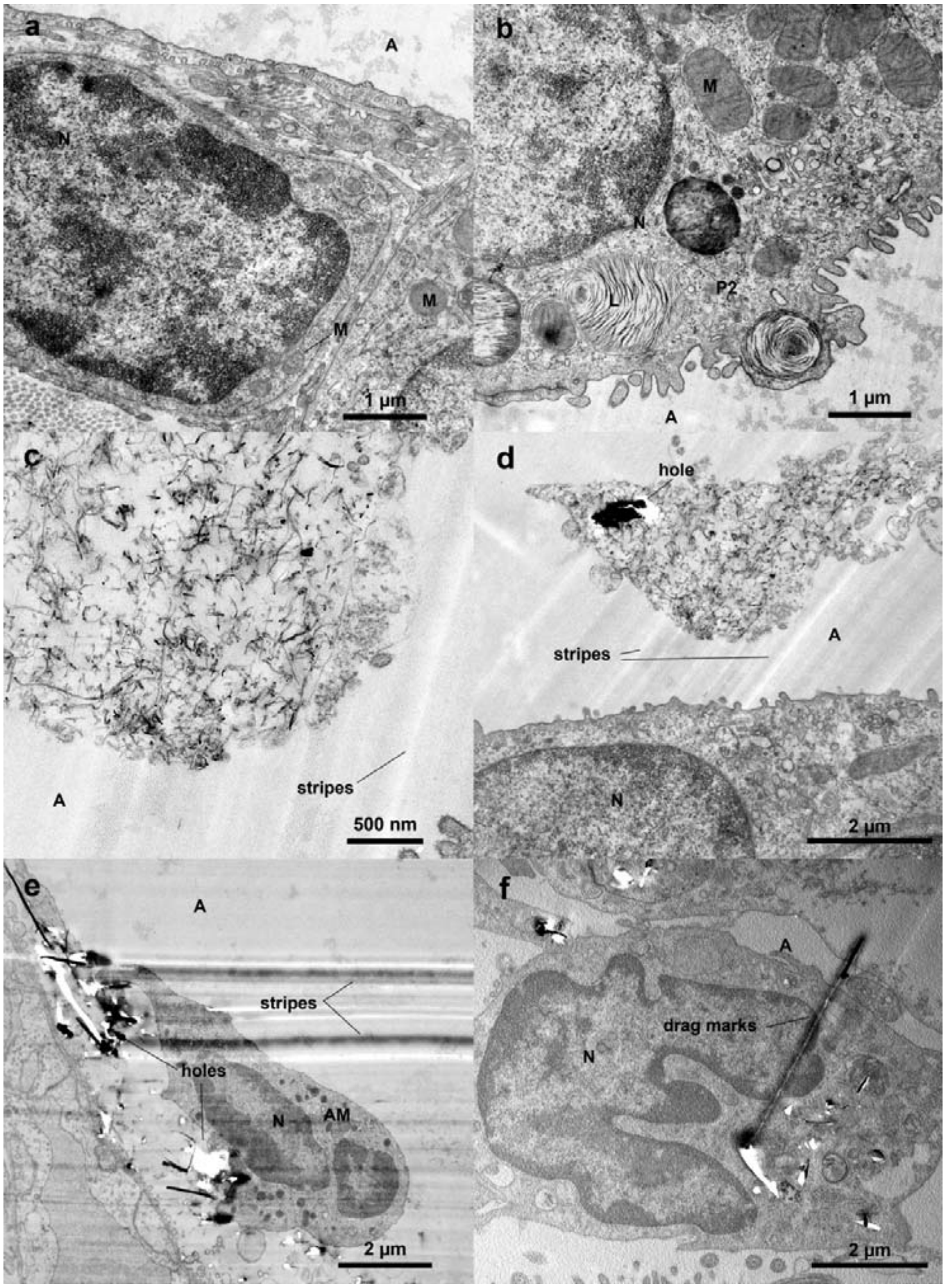

**Fig. S4.** SEM micrographs of ultramicrotomed epon blocks containing only the CNTs and no lung tissue

**a** image of the block containing **CNT**$_{Small}$. **b** SEM of the block containing only CNT$_{Large}$. CNTs were readily found on the microtomed surface of the pure CNT samples.

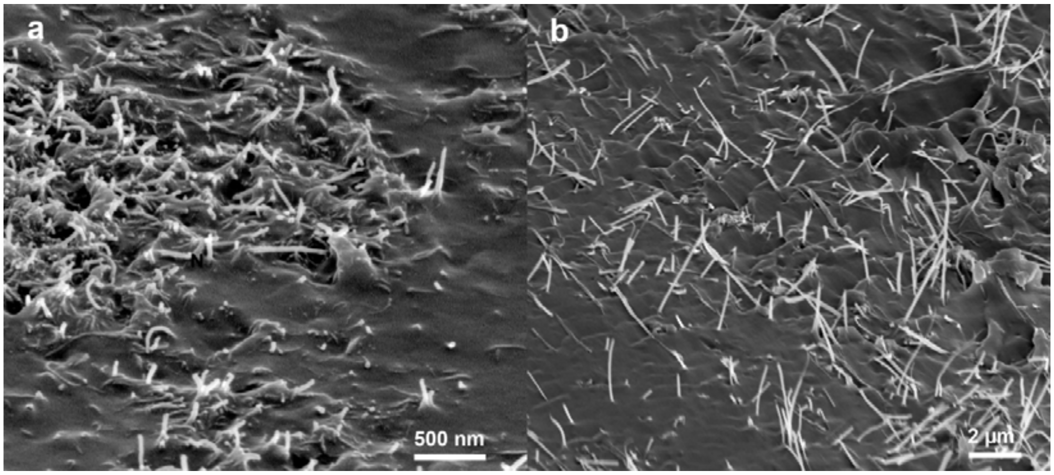

**Fig. S5.** FIB-SEM images of control tissue containing no CNTs

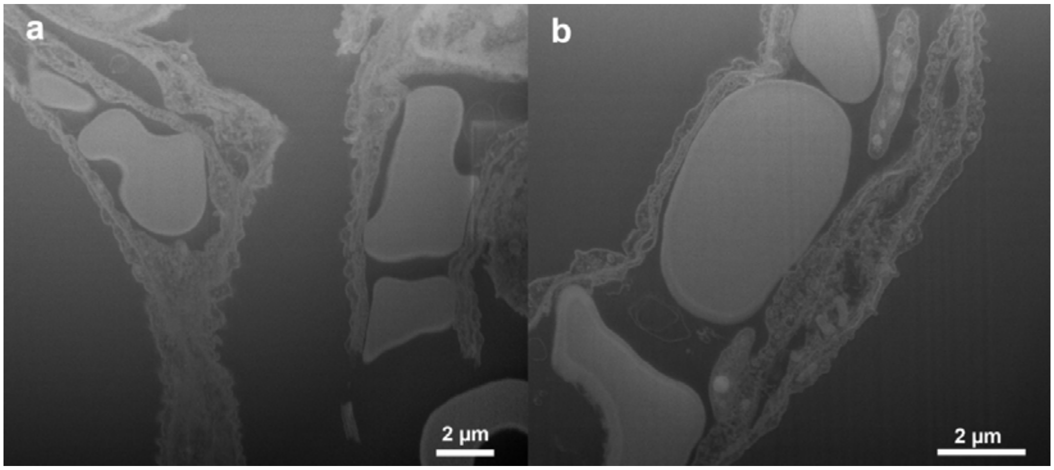

**Fig. S6.** SEM images of milling artefacts on samples with large CNTs

**a** Shows an extreme case of milling artefacts originating from within the embedded block and looking like protruding CNTs. **b** Closer view of CNTs protruding from the surface (black arrowheads) and one long CNT in the milling plane (white arrowheads). **c** When changing the view point to that of the ion beam, CNTs are in fact seen to protrude from the sample having not been completely removed by the ion beam (black arrowhead).

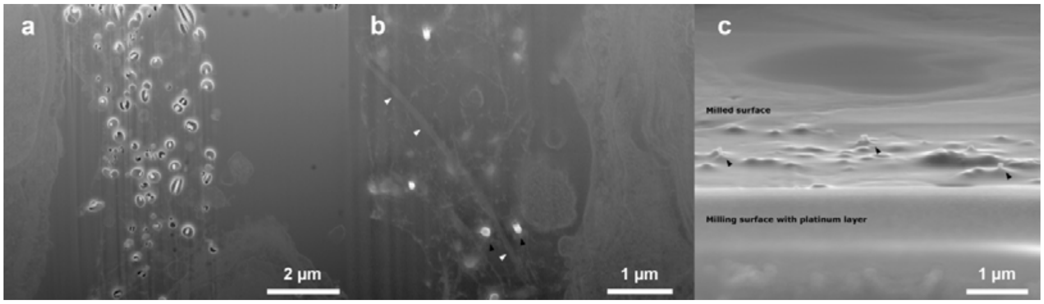

**Fig. S7.** Manual segmentation of CNTs in FIB-SEM images

**a** In this frame several individual CNTs are recognised (arrowheads) by their distinct parallel lines, neighbouring slides may reveal that more CNTs were partly visible in this frame. **b** Two of the CNTs are manually traced in Amira. This is done for multiple CNTs in all the slices. **c-d** Image of the starting and ending of a particular CNT. In each image slice (20 slices apart) the CNT fragment appears less than 500 nm long. **e** When the CNT (red) is traced in the 20 slices it now appears to be around 4 µm long and is possibly even longer as the CNT touches the edge of the field of view. Blue lines indicate previously traced CNTs.

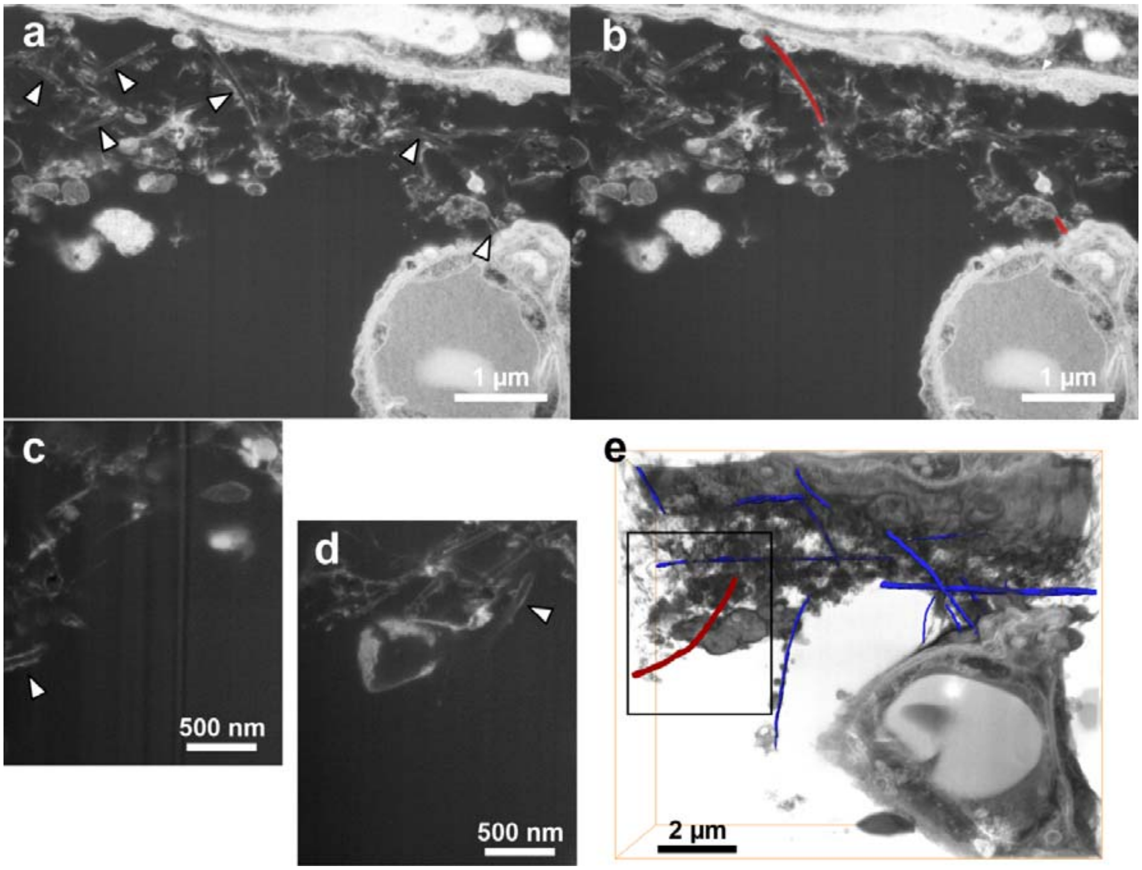


**Multimedia file:** http://dx.doi.org/10.1007/s00216-013-7566-x
